# Investigation of Oral Microbiome in Donkeys and the Effect of Dental Care on Oral Microbial Composition

**DOI:** 10.3390/ani10122245

**Published:** 2020-11-30

**Authors:** Yiping Zhu, Wuyan Jiang, Reed Holyoak, Bo Liu, Jing Li

**Affiliations:** 1 Equine Clinical Diagnostic Center, College of Veterinary Medicine, China Agricultural University, Beijing 100193, China; sy20203050871@cau.edu.cn (Y.Z.); jiangwyan@cau.edu.cn (W.J.); bliu2017@cau.edu.cn (B.L.); 2 College of Veterinary Medicine, Oklahoma State University, Stillwater, OK 74078, USA; reed.holyoak@okstate.edu

**Keywords:** oral microbiome, 16S rRNA, dental treatment, donkeys, China

## Abstract

**Simple Summary:**

Dental health in donkeys has long been neglected, even though it is quite common for them to have dental problems. Therefore, dental care, as basic as dental floating, can be a good start to improve their dental condition. Oral microbiome sequencing is a reliable way to reflect the oral health of animals. However, little is known on the effect of dental care on the oral microbiome of donkeys. Hence, a research project was undertaken to investigate the relationship between dental floating and oral microbial changes using a current sequencing technique. We found that the changes of the oral microbiome were not significant, probably due to the necessity of more specific and consistent treatment. However, the study provided an insight of the oral microbial composition and helped increase awareness of dental care in donkeys.

**Abstract:**

The objective of this study was to investigate the oral microbial composition of the donkey and whether basic dental treatment, such as dental floating, would make a difference to the oral microbial environment in donkeys with dental diseases using high-throughput bacterial 16S rRNA gene sequencing. Oral swab samples were collected from 14 donkeys with various dental abnormalities on day 0 (before treatment) and day 20 (twenty days after treatment). It is the first report focusing on the oral microbiome in donkeys with dental diseases and the impact of common dental procedures thereon. Identified in group Day 0 and group Day 20, respectively, were 60,439.6 and 58,579.1 operational taxonomic units (OTUs). Several taxa in Day 0 differed significantly from Day 20 at the phylum and genus levels, but no statistically significant difference was observed in richness and diversity of Day 0 and Day 20. The results also indicated that a larger-scale study focusing on healthy donkey oral microbiome, as well as the correlation of dental diseases and oral microbiomes at different time frames following more specific and consistent dental treatment, are warranted.

## 1. Introduction

Dental disease is a large welfare concern in donkeys [1]. The incidence of severe dental diseases such as diastema, overgrown teeth and periodontal diseases is high, [2] yet little attention has been paid to those conditions, and in fact, it is a common problem that most of donkeys do not receive dental examinations and preventative treatments regularly, either due to economic reasons or ignorance of dental care [3]. Diastema is the most common dental disease seen in donkeys [4] causing food entrapment and bacteria building up. The result is usually gingivitis and periodontitis and, in some cases, deep periodontal pockets [5] due to food mechanical irritation [6] and chemical destruction from bacterial infection [7]. In donkey farms in China, most donkeys are raised in large numbers and in high density. Additionally, donkeys are usually very stoic and good at hiding their discomfort, thereby eliminating clinical signs, even with severe dental diseases. As a result, their dental abnormalities are usually undiagnosed, causing severe pain, weight loss or even colic, until declining health leads to further examination and discovery of the dental disease [8]. Due to the high prevalence and negative impact of dental diseases in donkeys, dental care, especially the basic dental floating, is the most necessary treatment that can be provided routinely. There have been many studies conducted to investigate equine oral microbiome and what kinds of bacteria predominate or are associated with periodontal diseases in horses [8,9]; however, no report has focused on the donkey oral microbiome. Therefore, this study was intended to explore the oral microbial composition in donkeys and assess the impact of dental floating on the oral microbiome.

## 2. Materials and Methods

### 2.1. Animal Selection

The donkeys involved in this study were of the Liaoxi breed from a donkey farm in Northeast China. Donkeys in this farm were mainly female, used for milk and reproduction. No dental care had been performed on any of these animals before this study, and no antimicrobial medications had been administered in the previous 8 weeks to any of them selected for this study. Fourteen donkeys were randomly selected from a group of approximately 100 donkeys on Day 0. All were female, aged between 2 to 10 years old (mean age was 6). They were fed with mainly crop residue (corn stalk and millet stake) and supplemented with a small amount of homemade concentrate (maize, bran, soybean meal and salt) three times a day for years. None of the donkeys wore halters daily, only for special procedures—for example, the dental exam in this study. The examination started with a visual inspection of the skull symmetry and detection of ocular or nasal discharge. There was minimum-to-no obvious pain reactions such as head tossing noted while palpating and pressing the cheeks against the cheek teeth. Incisors were examined first to look for any abnormalities, such as brachygnathism, prognathism or occlusal surface abnormalities such as a slant, “frown” or extreme “smile”. Whereafter, they were each sedated, and the oral cavity was flushed with tap water. An incisor speculum (Hausmann speculum) was then applied to facilitate examination. Other tools used to detect dental abnormalities of cheek teeth included a head light, a dental mirror, a dental explorer and a periodontal probe. Dental disorders were recorded, including severe sharp enamel points, periodontal pockets, hooks, diastemata, shear mouth, step mouth, wave mouth, accentuated transverse ridges and caries. Following the dental examination, sample procurement from the oral cavity was conducted in all 14 donkeys.

### 2.2. Sample Collection

On Day 0, after a thorough oral exam, samples were collected by using a sterile swab rubbed around the gingival margin and on top of the gingival sulcus on the buccal side bilaterally with sufficient pressure [10]. Each swab was then individually placed in a sterile tube containing 1-mL sterile Tris-EDTA buffer solution and stored in ice until arrival at the laboratory and then deep-frozen at −20 °C awaiting analysis. After sampling, the mouth was rinsed off with a chlorohexidine solution, and dental floating was performed for each of the 14 donkeys to help reduce the severity of dental abnormalities and improve occlusion. Twenty days after the dental treatment, another dental examination was performed, and a second set of oral samples was collected and processed in the same way.

### 2.3. Sample Processing and DNA Extraction

All samples were maintained at ‒20 °C before DNA extraction. Sample DNA was obtained from the oral swabs using a Hi-Swab DNA Kit (Tiangen, Beijing, China) in accordance with the manufacturer’s instructions. The DNA concentration and purity were monitored by 1% agarose gel electrophoresis. According to the concentration, DNA was diluted to 1 ng/µL using sterile water.

### 2.4. 16S rRNA Sequencing

The V4 hyper-variable regions of the 16S rRNA genes were amplified by Novogene using the specific primers 515F (5′-3′ GTGCCAGCMGCCGCGGTAA) and 806R (5′-3′ GGACTACHVGGGTWTCTAAT), which target the V4 region of the bacterial 16S rRNA gene. Each PCR reaction was carried out in total volume of 30 µL with 15 µL of Phusion^®^ High-Fidelity PCR Master Mix (New England Biolabs, Ipswich, MA, USA); 0.2 µm of forward and reverse primers, 10-ng template DNA and ultrapure water. Thermal cycling consisted of initial denaturation at 98 °C for 60 s, 30 cycles of denaturation at 98 °C for 10 s, 50 °C for 30 s, 72 °C for 30 s and one final cycle of 72 °C for 300 s.

### 2.5. Statistical Analysis

For α-diversity, the richness of samples representing the number of different bacterial taxa in each group was assessed using the number of observed operational taxonomic units (OTUs) and the Shannon index. In addition, the Simpson index was used for assessing evenness. The Illumina-sequenced paired-end reads were merged by using FLASH (V1.2.7). Sequences were processed by using the QIIME (V1.9.1, Quantitative Insights Into Microbial Ecology) software package for quality filtering and construction of the OTUs [11]. Sequences with ≥97% similarity were assigned to the same OTUs. The RDP (ribosomal database project) classifier was used to annotate the taxonomic information for each representative sequence.

The Kolmogorov-Smirnov test and the Shapiro-Wilk test were used to assess continuous data for normal distribution. *T*-test for paired samples was applied for the comparisons of the Shannon index. β-diversity analysis was performed to compare samples. Principal component analysis (PCA) was used to evaluate the general distribution of the resulting bacterial community composition. The linear discriminant analysis effect size (LEfSe) pipeline was also applied.

Paired *t*-test (normally distributed values) and Mann-Whitney U test (non-normally distributed values) were used for the statistical evaluation of continuous variables.

## 3. Results

### 3.1. Clinical Findings

On Day 0, all 14 donkeys in this study had mild-to-severe sharp enamel points and a varied degree of periodontal pockets (Table 1). According to the incisor abnormalities, only four (29%) donkeys had mild brachygnathism. No other disorders, such as smile, slant or prognathism, were noted. In this study group, 50% of the donkeys had hooks and diastema. Additionally, nearly half of the donkeys (43%) suffered from oral ulceration, and fibrously healed ulcers were observed in some of the donkeys. Four (29%) donkeys had a transverse ridge, all of which also had diastema. The occurrence of wave mouth (7%), step (14%), shear mouth (7%) and caries (14%) constituted the remainder of the dental disorders in this study. On Day 20, there were no donkeys with sharp enamel points or hooks. The diastema found in the seven donkeys on Day 0 was still there; however, less food was trapped, especially those with concurrent transverse ridges on Day 0. Most of the ulcers noted on Day 0 started healing, and some were almost healed. No significant changes were noted on periodontal pocket depths. Steps, wave mouth and shear mouth were still noted, but the evenness was improved.

### 3.2. Microbiome Profile Analysis

**Figure 1:**
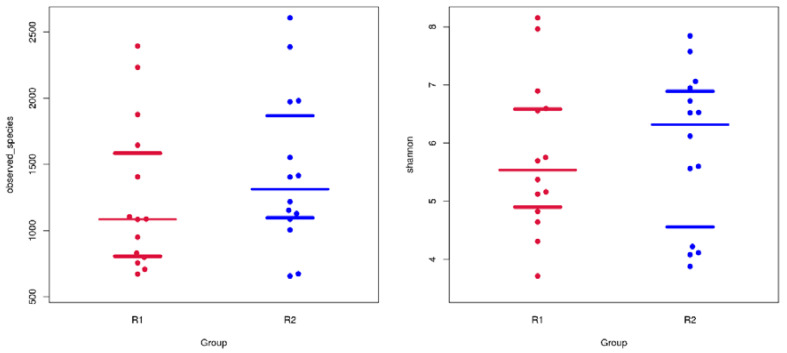
Observed operational taxonomic units (OTUs) (**left**) and Shannon index (**right**). Red color indicates the Day 0 group, while blue indicates the Day 20 group.

The mean values of the sequences were 60,439.6 and 58,579.1 (Mann-Whitney U test, *P* = 0.401) from samples collected on Day 0 and Day 20, respectively. The microbial richness and diversity were evaluated with α-diversity indexes, including the Chao1 index, observed OTUs, Shannon index and Simpson index. The richer bacterial microbiomes are indicated by a higher Chao1 index or observed OTUs, while more diverse bacterial microbiomes are indicated by a higher Shannon index or lower Simpson index. There was no significant difference of the mean number of OTUs (paired *t*-test, *P* = 0.414), Chao1 index (*P* = 0.291), Shannon index (*P* = 0.779) and observed OTUs (*P* = 0.306) (Figure 1) between the two groups, meaning the richness of Day 0 and Day 20 were not significantly different.

### 3.3. Compositional Analysis between the Groups

**Figure 2:**
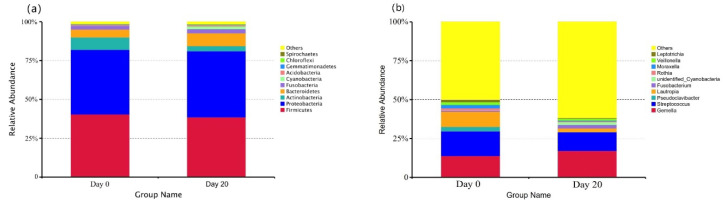
(**a**) Microbial composition of Day 0 and Day 20 at the phylum level. (**b**) Microbial composition of Day 0 and Day 20 at the genus level.

When the taxonomic abundance of the two groups of sequences was compared at the phylum level, the microbial community of the two groups featured a similar profile. The relative abundance of the top nine phyla in both groups are displayed in Figure 2a, which are *Firmicutes*, *Proteobacteria*, *Actinobacteria*, *Bacteriodetes*, *Fusobacteria*, *Cyanobacteria*, *Acidobacteria, Gemmatimonadetes, Chloroflexi* and *Spirochaetes*. Among these phyla, each of the first five phyla (*Firmicutes*, *Proteobacteria*, *Actinobacteria*, *Bacteriodetes* and *Fusobacteria*) accounted for >1% of the total sequences amongst both groups. *Firmicutes* and *Proteobacteria* were the most common bacterial phyla in the Day 0 group and Day 20 group, which accounted for 81.1–81.9% (Figure 2a). *Cyanobacteria* was 1.8% on Day 20, which increased significantly compared to 0.06% on Day 0 (*P* = 0.004). Actinobacteria had significantly lower relative abundance (3.3%) on Day 20 after dental treatment compared to the data (8.8%) on Day 0 (*P* = 0.035).

There were also some differences of the taxonomic abundance at the genus level (Figure 2b). Eight hundred and ninety-one operational taxonomic units (OTUs) were identified at the genus level. The differences of taxonomic abundance were more notable from the two groups, and the shifts of the bacterial taxa were more significant at this level. *Gemella* and *Streptococcus* were the most common bacterial genera in both groups. Higher levels of *Pseudoclavibacter* (2.97%), *Lautropia* (9.74%), *Moraxella* (2.17%), *Leptotrichia* (1.64%) and *Rothia* (1.35%) were found in the Day 0 group.

**Figure 3:**
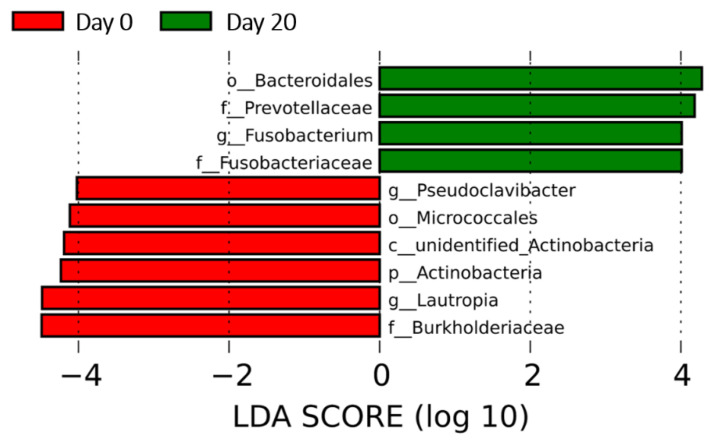
Linear discriminant analysis (LDA) effect size analysis indicating the bacterial taxa (genus or higher level) most associated with the Day 0 group and Day 20 group, respectively. Only 10 taxa that had LDA scores above 2 were shown. The taxa were ranked by the effect size in linear discriminant analysis effect size (LEfSe).

Β-diversity was accessed by a principal coordinate analysis (PCoA), which did not show statistic difference at the phylum level, while there was a greater overall variance in the Day 20 group than in the Day 0 group (Figure S1). The linear discriminant analysis (LDA) effect size (LEfSe) was applied, and ten discriminant taxa (Figure 3) at the genus or higher levels were found significantly different between Day 0 and Day 20 (LDA score >2, *P* < 0.05). The genera found most associated with the Day 0 group were *Lautropia* and *Pseudoclavibacter*, while *Fusobacterium* was the genus most associated with the Day 20 group. *Burkholderiaceae* and *Bacteoidales* yielded the highest LDA scores among the periodontal biomarkers in the Day 0 group and Day 20 group, respectively.

## 4. Discussion

This is the first report investigating the oral microbiome in donkeys and comparing the microbial changes before and after dental floating. All donkeys in this study were female due to the production purpose of this farm. There has been no report of the correlation of sex and oral microbial composition, while gut microbiota variance has exhibited an association with sex in other species [12], including horses, through poorly understood mechanisms [13]. Therefore, the oral cavity, a part of the digestive system, may also have different microbiomes between male and female donkeys. A study carried out in Mexico involving 203 donkeys (69% males and 31% females) showed that sex was significantly associated with dental diseases, probably due to the utility of nosebands in male donkeys causing more traumatic dental and soft tissue buccal injuries. [5]. Hence, it is reasonable to consider that the individual’s sex may have some influence on the oral microbial composition. While our study was not confounded by animal sex influence, further investigation in this area is warranted.

The feeding pattern of donkeys in this farm only allowed them to chew for short periods of time during the day, which may cause the teeth wearing down inadequately and unevenly [14]. Therefore, it could lead to an increased risk of dental disorders such as sharp enamel points. There was also much evidence showing that fiber functioned as a mechanical force on dental calculus in some animal studies [15,16], which could impact the oral microbiome associated with different dental conditions. Additionally, carbohydrates, lipids and minerals, as well as vitamins, were all factors that had exhibited correlations with some bacterial taxa in human studies [17,18]. Therefore, further investigations targeting the diet composition and oral microbiome of donkeys will help to promote scientific diets for them and be beneficial to their welfare.

The environment is another popular variable input while studying a microbiome, yet no report regarding the association of environmental changes and oral microbiota composition in animals has been published. However, environmental factors such as husbandry conditions in terms of stable types and grazing periods have been proven to have a great influence on the richness and diversity of gut microbiota in horses [19]. Derived from these results, we hypothesized that oral microbiota may change in different environmental conditions. Since, in this study, all donkeys were from the same farm, a controlled study designed in different local donkey farms would be helpful to investigate this hypothesis.

Oral microbiome changes have been associated with dental disease, especially periodontal diseases in humans and many animal species, including horses [9,11] dogs [20], cats [21] and cattle [22], but not yet in donkeys suffering from dental abnormalities. Dental floating, the most common dental treatment, plays an important role in dental disease control within farm-raised donkeys. Therefore, it is meaningful to know if there is an impact of dental floating on the oral microbiome in donkeys so that dental care can be conducted in a more scientific way in the future.

The donkeys selected in our study had severe sharp enamel points prevalently, which are especially common in younger donkeys [1], and periodontal pockets, which are commonly associated with diastema in horses [23]. In equids, diastema is considered related to periodontal diseases, such as periodontitis [24], which usually further impacts the oral microbiome [23]. Diastema generally requires a series of dental treatments, similarly to other severe dental abnormalities seen in this study, including shear mouth, wave mouth and step mouth. Dental care, especially dental floating, is usually the fundamental step of treating most dental disorders and could also improve those dental disorders to some extent. For example, decreasing the amount of food trapped into the diastema by reducing the transverse ridges on the opposite site [25]; therefore, we proposed that it might subsequently lead to some microbial changes before (Day 0) and after (Day 20) our dental treatment.

In the current study, 16S rRNA sequencing revealed that the composition of the oral microbiome of the two groups (Day 0 and Day 20) were not significantly different in richness and diversity, indicating that dental floating did not render many changes at the microbial level. However, the relative differences indicated a shift in relative abundance, indicating a reassortment in dominance. Hence, more specific and consistent dental treatments on dental disorders may yield more optimal outcomes, and more studies focusing on these topics are worthwhile for the better control and improvement of donkey dental conditions. In previous research using the same 16S rRNA sequencing technique, *Actinobacteria*, *Bacteriodetes, Firmicutes, Proteobacterio* and *Spirochaetes*, which were found predominant in both groups at the phylum level in this study, were also commonly found in the mouths of healthy humans [26], equines [5] and canines [27], with slightly different proportions between the oral microbiomes. This suggests that the oral microbiome in the donkey share some similarities with those of humans and other animals. *Firmicutes*, the most common bacteria phyla in this study, was also a common inhabitant of the oral cavity, especially in the subgingival area. However, some studies focusing on oral microbiomes in dogs and cats with periodontitis showed that it was one of the predominant phyla in the periodontitis group. *Firmicutes* was also abundant in horse subgingival plaque, likely acting as an opportunistic pathogen and contributing to periodontitis [8]. *Proteobacteria*, the second-highest bacterial phylum in this study, was also abundant in horse subgingival plaques [9]. Further evaluations with specific sampling may provide more insight on whether they are associated with periodontal diseases or not in donkeys.

*Gemella* spp. and *Streptococcus* spp. were the most common bacterial genera in this study, as well as in many animals in other studies [28]. *Gemella* was slightly more predominant in the Day 20 group, while *Streptococcus* was slightly lower in the Day 20 group. *Gemella* has been shown more prominent in healthy horses [8], and *Streptococcus* has been reported to be the most common genera of *Firmicutes* in the healthy human oral microbiome [29], but indeed, some species have been associated with active caries [30]. Dental floating may have contributed to the increase of *Gemella* in the Day 20 group but not enough for notable changes.

*Fusobacterium*, an obligate, anaerobic bacterium, is considered as a common oral niche inhabitant in humans and some other animals, including horses [31]. In this study, it was shown to be most associated with the Day 20 group, while *Lautropia* and *Pseudoclavibacter* were the genera most associated with Day 0 in LEfSe. These changes were likely an indication of an expansion of the lesser phyla allowed by suppression of the more dominant microflora present in Day 0. Its population can increase significantly as an opportunistic pathogen in oral disease and has been cultured from a horse and a donkey with dental abscesses [32]. Some specific species of *Fusobacterium*, such as *F. nucleatum*, have been associated with periodontal diseases in many animals, including dogs, cats and horses [33,34,35]. However, in this study, its population did not show a dramatic increase. The sampling specifically from periodontal pockets from donkeys only with periodontitis may help reveal better associations with dental treatment. Additionally, there was a relatively rare bacterial taxon at the genus level identified on Day 0 that was *Pseudoclavibacter*, belonging to the *Actinobacteria* phylum. So far, nothing has been reported about this bacterial genus in oral microbiome studies either in humans or in animals. It was not identified in the Day 20 group and only associated with the Day 0 group according to the LDA score, which may indicate some associations between this bacteria genus and donkey dental diseases. Further studies involving more individuals will be helpful to decide whether *Pseudoclavibacter* is specific in donkeys and associated with dental diseases or merely an environmental contaminant.

## 5. Conclusions

In conclusion, this is the first report of the donkey oral microbiome in association with basic dental treatment. Our results did not reveal significant differences of the oral microbial composition in selected donkeys; however, there were bacterial genera showing significant differences between the Day 0 and Day 20 groups, as well as a strong association to each group. These shifts of relative diversity and dominance indicated a reassortment relative to the dental treatment. Furthermore, our study revealed that the donkeys shared many similarities with other animals at the phylum and genus levels. Additional studies to investigate the donkey oral microbiome in healthy conditions, as well as its associations with specific types of oral diseases, such as periodontitis and diastema, by selecting samples specifically will promote an understanding of the impact of these diseases on donkey dental health. It will also build up a basis for studying the influences of other potential factors, including diet, feeding pattern and farm environment on the donkeys’ dental condition, so that more scientific raising methods can be established to improve their quality of life.

## Figures and Tables

**Table 1:** Prevalence of dental disorders (*n* = 14 donkeys).

Dental Disorders	Number of Donkeys	Prevalence
Periodontal pockets (2–5 mm)	14	100%
Sharp enamel points	14	100%
Hooks	7	50%
Diastemata	7	50%
Ulcers	6	43%
Disorders in the occlusal surface	5	36%
Transverse ridge	4	29%
Brachygnathism	4	29%
Caries	2	14%
Step	2	14%
Shear mouth	1	7%
Wave mouth	1	7%

## Supplementary Materials

The following are available online at https://www.mdpi.com/2076-2615/10/12/2245/s1, Figure S1: Principal coordinates analysis (PCoA) plot showing overlap of oral microbiomes in group Day 0 and group Day 20.

## References

[1] Burden F., Du Toit N., Thiemann A. (2013). Nutrition and dental care of donkeys. Practice.

[2] Du Toit N., Gallagher J., Burden F.A., Dixon P.M. (2010). Post mortem survey of dental disorders in 349 donkeys from an ages population (2005–2006). Part 1: Prevelance of specific dental disorders. Equine Vet. J..

[3] Rodrigues J.B., Lilly G. (2019). Dental disorders of Donkey. Vet. Clin. N. Am. Equine Pract..

[4] Du Toit N., Dixon P.M. (2012). Common dental disorders in the donkey. Equine Vet. Educ..

[5] Du Toit N., Burden F.A., Dixon P.M. (2008). Clinical dental findings in 203 working donkeys in Mexico. Vet. J..

[6] Du Toit N., Burden F.A., Baedt L.G., Shaw D.J., Dixon P.M. (2009). Dimensions of Diastemata and Associated Periodontal Food Pockets in Donkey Cheek Teeth. J. Vet. Dent..

[7] Walker H., Chinn E., Holmes S., Barwisemunro L., Bobertson V., Bradley S., Shaw D.J., Dixon P.M. (2012). Prevalence and some clinical characteristics of equine cheek teeth diastemata in 471 horses examined in a UK first-opinion equine practice (2008 to 2009). Vet. Rec..

[8] Du Toit N., Burden F.A., Dixon P.M. (2009). Clinical dental examination of 357 donkeys in the UK. Part 1: Prevalence of dental disorders. Equine Vet. J..

[9] Gao W., Chan Y., You M., Lacap-Bugler D.C., Leung W.K., Watt R.M. (2015). In-depth snapshot of the equine subgingival microbiome. Microb. Pathog..

[10] Kennedy R., Lappin D.F., Dixon P.M., Buijs M.J., Zaura E., Crielaard W., O’Donnell L., Bennett D., Brandt B.W., Riggio M.P. (2016). The microbiome associated with equine periodontitis and oral health. Vet. Res..

[11] Caporaso J.G., Kuczynski J., Stombaugh J., Bittinger K., Bushman F.D., Costello E.K., Fierer N., Pena A.G., Goodrich J.K., Gordon J.I. (2010). QIIME allows analysis of high-throughput community sequencing data. Nat. Methods.

[12] Freire A.C., Basit A.W., Choudhary R., Piong C.W., Merchant H.A. (2011). Does sex matter? The influence of gender on gastrointestinal physiology and drug delivery. Int. J. Pharm..

[13] Mshelia E.S., Adamu L., Wakil Y., Turaki U.A., Gulani I.A., Musa J. (2018). The association between gut microbiome, sex, age and body condition scores of horses in Maiduguri and its environs. Microb. Pathog..

[14] Feeding Practices May Impact Horse Dental Health. https://ker.com/equinews/feeding-practices-may-impact-horse-dental-health/.

[15] Van A., Beynen A.C., Altena F.V., Visser E.A. (2010). Beneficial effect of a cellulose-containing chew treat on canine periodontal disease in a double-blind, placebo-controlled trial. Am. J. Anim. Vet. Sci..

[16] Logan E.I. Oral cleansing by dietary means: Results of six-months studies. Proceedings of the Companion Animal Oral Health Conference.

[17] Murtaza N., Burke L.M., Vlahovich N., Charlesson B., O’Neill H.M., Ross M.L., Campbell K.L., Krause L., Morrison M. (2019). Analysis of the effects of dietary pattern on the oral microbiome of elite endurance athletes. Nutrients.

[18] Kato I., Vasquez A., Moyerbrailean G., Land S., Djuric Z., Sun J., Lin H., Ram J.L. (2017). Nutritional correlates of human oral microbiome. J. Am. Coll. Nutr..

[19] Kaiser-Thom S., Hilty M., Gerber V. (2020). Effects of hypersensitivity disorders and environmental factors on the equine intestinal microbiota. Vet. Q..

[20] Ruparell A., Iuni T., Staunton R., Wallis C., Deusch O., Holcombe L.J. (2020). The canine oral microbiome: Variation in bacterial populations across different niches. BMC Med..

[21] Dewhirst F.E., Klein E.A., Bennett M.L., Croft J.M., Harris S.J., Marshall-Jones Z.V. (2015). The feline oral microbiome: A provisional 16S rRNA gene based on taxonomy with full-length reference sequences. Vet. Microbiol..

[22] Borsanelli A.C., Lappin D.F., Viora L., Bennett D., Dutra I.S., Brandt B.W., Riggio M.P. (2018). Microbiomes associated with bovine periodontitis and oral health. Vet. Microbiol..

[23] Chinkangsadarn T. (2015). Clinical and Microbiological Aspects of Periodontal Disease in Horses in South-East Queensland, Australia. Ph.D. Thesis.

[24] Kennedy R.S., Dixon P.M. (2018). The aetiopathogenesis of equine periodontal disease-a fresh perspective. Equine Vet. Educ..

[25] Collins N.M., Dixon P.M. (2005). Diagnosis and management of equine diastema. Clin. Tech. Equine Pract..

[26] Palmer R.J. (2014). Composition and development of oral bacterial communities. Periodontology.

[27] Holcombe L.J., Patel N., Colyer A., Deusch O., O’ Flynn C., Harris S. (2014). Early canine plaque biofilms: Characterization of key bacterial interactions involved in initial colonization of enamel. PLoS ONE.

[28] Bik E.M., Long C.D., Armitage G.C., Loomer P., Emerson J., Mongodin E.F., Nelson K.E., Gill S.R., Fraser-Liggett C.M., Relman D.A. (2010). Bacterial diversity in the oral cavity of 10 healthy individuals. ISME J..

[29] McLean J.S. (2014). Advancements toward a systems level understanding of the human oral microbiome. Front. Cell. Infect. Microbiol..

[30] Dewhirst F.E., Chen T., Izard J., Paster B.J., Tanner A.C.R., Yu W., Lakshmanan A., Wade W.G. (2010). The human oral microbiome. J. Bacteriol..

[31] Darrene L.N., Cecile B. (2016). Experimental models of oral biofilms developed on inert substrates: A review of the literature. BioMed Res. Intern..

[32] Takada K., Hayashi K., Sato Y., Hirasawa M. (2010). Prevotella dentasini sp. nov., a black-pigmented species isolated from the oral cavity of donkeys. Int. J. Syst..

[33] Mallonee D.H., Harvey C.E., Venner M., Hammond B.F. (1988). Bacteriology of periodontal disease in the cat. Arch. Oral Biol..

[34] Sturgeon A., Pinder S.L., Costa M.C., Weese J.S. (2014). Characterization of the oral microbiota of healthy cats using next-generation sequencing. Vet. J..

[35] Sturgeon A., Stull J.W., Costa M.C., Weese J.S. (2013). Metagenomic analysis of the canine oral cavity as revealed by high-throughput pyrosequencing of the 16S rRNA gene. Vet. Microbiol..

