# Supplementary figures and images for: Investigation of Oral Microbiome in Donkeys and the Effect of Dental Care on Oral Microbial Composition
Source: Animals (Basel). 2020 Nov 30;10(12):2245. doi: 10.3390/ani10122245 (PMC7761040; doi:10.3390/ani10122245)

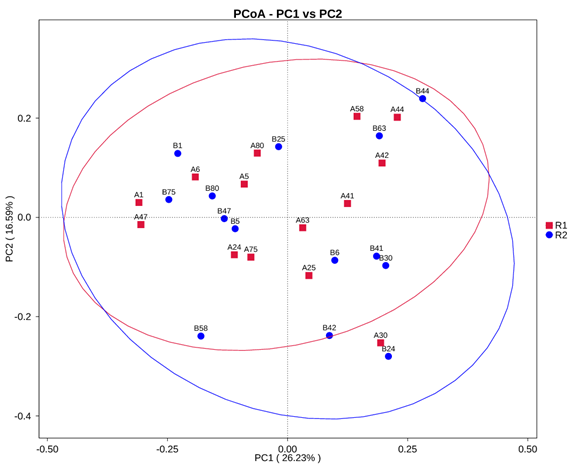

Supplement: Supplementary file 1 [file animals-10-02245-s001.zip › animals-1018541-supplementary.png]
